# Waiting time in the premilking holding pen and subsequent lying and walking behaviors of Holstein cows

**DOI:** 10.3168/jdsc.2021-0205

**Published:** 2022-04-26

**Authors:** D. Manríquez, S. Zúñiga, S. Paudyal, G. Solano, P.J. Pinedo

**Affiliations:** 1 Department of Animal Sciences, Colorado State University, Fort Collins 80523-1171; 2 Department of Animal Sciences, Texas A&M University, College Station 77843

## Abstract

The objective of this study was to assess the effect of waiting time (WT) in the premilking holding pen on the subsequent lying and walking behaviors of Holstein cows. A total of 108 cows (multiparous n = 95; primiparous n = 13), milked 3 times/d [AM (0700 h), PM (1500 h), and nighttime (2300 h)] were monitored for individual WT, which was defined as the time elapsed between the entrance of the first cow to the milking stall in a rotary milking system and the entrance of each subsequent cow housed in the same milking pen. Data for lying time (min), standing time (min), lying bouts (no.), and walking behavior (no. of steps) in 15-min intervals were collected using a commercial 3-dimensional accelerometer affixed on the hind left leg of each cow from early to mid lactation. Mean values (min/h, no./h) for each behavior calculated between consecutive milkings were compared among 3 WT categories: cows with WT ≤30 min (WT1), WT 30 to 60 min (WT2), and WT >60 min (WT3). The overall mean (SD) of WT during the study period was 31.2 (23.4) min, and the distribution of WT1, WT2, and WT3 in all cows through the monitoring period was 50.9, 43.3, and 5.7%, respectively. No significant effects were established for overall WT on any of the behaviors under analysis. However, subsequent analysis by milking shift indicated that, during the period following the night milking, cows in WT1 exhibited the shortest lying time, whereas cows in WT3 had the greatest number of lying bouts. No effect of WT category was observed on the number of steps after any of the milking shifts. Results of this study indicate a moderate effect of WT on lying behavior after the nighttime milking. Variables such as temperature-humidity index, parity, and time of day affected cow behavior and should be considered when evaluating the impact of routine management practices, such as milking, on commercial farms.

## Highlights

• Automated sensors allow monitoring of behavioral and physiological variables from large numbers of dairy cows, opening new possibilities for research.
• Waiting time in the premilking holding area can vary greatly among dairies and individual cows and could affect the time budget of cows.
• We found a moderate effect of waiting time before milking on lying time and lying bouts after the night milking.

## Graphical Abstract

**Figure:**
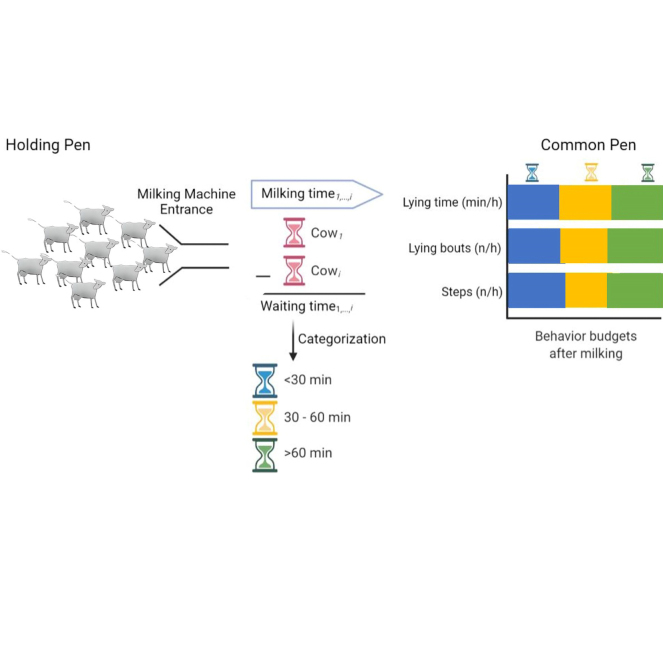
**Summary:** The objective of this study was to assess the effect of waiting time (WT) in the premilking holding pen on the subsequent lying and locomotion behaviors of Holstein cows. We analyzed the effect of WT on lying time, number of lying bouts, and number of steps using a commercial 3-dimensional accelerometer affixed to the hind leg. Mean values for each behavior between consecutive milkings were compared among 3 WT categories (short, intermediate, and long). No significant effects were detected for overall WT on any of the behaviors. However, analysis by milking shift indicated that, during the period following the night milking, cows subjected to short WT had the lowest lying time, whereas cows in the long WT category had the greatest number of lying bouts. No effect of WT category was observed for number of steps after any of the milking shifts. Variables such as parity, temperature-humidity index, and time of day affected cow behavior and should be considered when evaluating the effect of routine management of milking on commercial farms.

The continuous improvement of automated sensors to monitor behavioral and physiological variables from large numbers of dairy cows has opened new possibilities for improved management at the individual and herd levels. A variety of devices affixed to the cow's body can measure rumination (Gusterer et al., 2020; Cocco et al., 2021), eating time (Pereira et al., 2018; Dittrich et al., 2019), and resting and locomotion activity (Yunta et al., 2012; Reith et al., 2014; Weigele et al., 2018). Generally, these devices use 3-dimensional accelerometers that associate specific movements with activities performed by the cows (Weigele et al., 2018; Gusterer et al., 2020). Research indicates that most of them can be used accurately to estimate behaviors such as rumination and locomotion (Borchers et al., 2016; Pereira et al., 2018).

In practical terms, monitoring cow behavior has been integrated with farm health and reproductive programs, and deviations from baseline behaviors are considered indicative of concurrent metabolic disease (Liboreiro et al., 2015), expression of estrus (Mottram, 2016), approaching calving (Ouellet et al., 2016), and lameness (Yunta et al., 2012). Nonetheless, data generated by remote sensor devices offer opportunities to assess the effect of routine management practices on the behavior of dairy cows. Such measurements could be used to evaluate the potential impact of these practices on cow welfare, health, and performance.

Milking is the core of the daily routine of dairy cows, and it is plausible to anticipate that this activity might affect not only cows' time budget but also their welfare and health, depending on factors such as milking parlor design, pen stocking density, cow handling, and milking frequency.

The time used to milk a group of cows can vary greatly among dairies, and the milking routine has an inherent premilking waiting time (**WT**) that, for individual cows, can range from a few minutes to over an hour. Notably, cows establish hierarchies in the milking order within the group; consequently, individual cows may be consistently exposed to longer WT (McVey et al., 2020). This disparity in WT within a group of cows could affect the cows' time budgets between milkings, as cows subject to different timespans away from their pen might allocate their time for specific behaviors differently.

Individual lying and walking information from wearable sensors allow for the assessment of potential associations with variable WT that could result in negative outcomes, such as insufficient rest or feed intake. In this study, we hypothesized that WT in the premilking holding area affects the subsequent time budget of cows. Therefore, the objective of this study was to assess the effect of WT on subsequent behaviors: lying time (**LT**, min/h), lying bouts (**LB**, no./h), and walking [steps (**STP**, no./h)] of Holstein cows.

A total of 108 [multiparous (**MP**) n = 95; primiparous (**PP**) n = 13] lactating Holstein cows housed in an organic certified dairy farm located in northern Colorado were randomly enrolled within 20 DIM for a prospective single cohort study (Colorado State University, IACUC protocol ID: 17–7665A). The sample size was limited to the available number of sensors. Selected cows calved between November 22, 2017, and January 13, 2018, and were monitored until June 8, 2018. Study cows were affixed with a triaxial accelerometer (IceTag, IceRobotics Ltd.) on the lateral side of the left hind leg. Monitoring consisted of measurements of WT and subsequent lying time (min), standing time (min), lying bouts (no.), and steps (no.) between consecutive milkings. The accelerometers provided individual readings at a sampling rate of 15 min that were stored in .csv files and subsequently standardized by hour as minutes/hour (LT and standing time) and number/hour (LB and STP). As LT and standing time are mutually exclusive and complementary, STP was omitted from the analysis.

The study farm milked 1,700 cows 3 times daily in a 60-stall milking carousel. The 3 daily milkings were distributed in the morning (**AM**; 0700 h), afternoon (**PM**; 1500 h), and night (2300 h) schedules. The holding area of the milking parlor had capacity for 350 cows. The distribution of MP and PP cows in the study farm was 70% and 30%, respectively. During the study period, MP and PP cows were housed in the same pen. Cows were maintained in a 350-freestall barn provided with sand bedding, headlocks (75 cm of feed bunk space/cow), and access to an outdoor dry lot and to ad libitum water. The stocking rate in the fresh pen was 80% and this rate was maintained around 100% in the subsequent groups. Freestall cleaning and manure removal from the barn's alleys were performed twice daily during the morning and night milkings, and scraping of the dry lots was completed every other day. The dry lots remained open throughout the winter. Under extreme weather conditions, the access to the dry lots could be temporarily restricted. A TMR was fed twice daily to meet or exceed the nutritional requirements for lactating Holstein cows producing 30 kg/d of milk (3.5% fat and 3.1% true protein; NRC, 2001).

The effect of premilking WT on subsequent lying and walking behaviors was the main factor assessed in this study. Waiting time was calculated as the time between the entrance of the first cow to the milking stall in a rotary milking system and the entrance of each subsequent cow housed in the same milking pen. Considering the distribution of WT across the study period, this variable was categorized into 3 levels for each cow at every milking as follows: WT1 = WT ≤30 min; WT2 = WT 30 to 60 min; and WT3 = WT >60 min. The cut-off time for WT3 was intended to produce a group that would allow for testing an extreme WT.

Other covariates assessed were milking shift (AM, PM, and night), parity (PP and MP), concurrent health disorders, and temperature-humidity index (**THI**) during milking. Information about health disorders (milk fever, metritis, endometritis, clinical mastitis, digestive disorders, and respiratory disease) was retrieved from the on-farm recording software (PCDART, Dairy Records Management Systems). Based on this information, a health status category (sick = 1, healthy = 0) was created considering the diagnosis date. Estrous activity was not recorded unless cows were submitted for AI after the voluntary waiting period. Considering this limitation and to avoid inconsistencies, this variable was excluded from the analysis. Finally, sensors (HOBO UX100-011, Onset Computer Corp.) located in the freestall barn and in the holding area of the milking parlors measured the ambient temperature (T; °C) and relative humidity (RH). The THI was calculated using the following equation (Kendall et al., 2008):

THI = (1.8 × T + 32) – [(0.55 – 0.0055 × RH) × (1.8 × T – 26)].

As most of the monitoring period was during the cold season, the average daily values of THI were classified as low (THI ≤40) or high (THI >40).

Individual milking start times had a timestamp (formatted mm/dd/yyyy hh:mm:ss) from which we calculated the time between milkings and the budget of each locomotion behavior between milkings for each study cow. PROC SQL of SAS 9.4 (SAS Institute Inc.) was used to merge the time between milking and the subsequent locomotion behavioral values to each cow's ID on every milking. The grouping criteria were cow ID and the timestamp. From the merged data set, the timestamp values of the time between milking and LT were transformed to numeric format in Excel (Microsoft Corp.) for statistical analyses. A master data set was created to merge behavioral and milking time data with data from parity, health, and THI. Finally, LT, STP, and LB values were standardized as hourly rates to account for the variation in time available for each cow between 2 subsequent milkings.

Descriptive analyses for lying and walking behaviors were performed using PROC MEANS and PROC FREQ. Least squares means (**LSM**) were calculated using PROC MIXED for repeated measures. As cows are exposed to different events depending on the time of day, the overall analyses were followed by analyses separated by milking shift (Figure 1, Figure 2). The covariates in the initial models included WT, milking shift, parity, health category, and THI category. Additionally, the interactions between WT and milking shift and between WT and parity were tested. Backward elimination was used to select the final model of each lying and walking behavior. Covariance parameters were adjusted within each milking shift using the group option. The LSM of the covariates of interest were compared and *P*-values were adjusted using the Tukey test. Statistical significance was determined at *P* < 0.05 and controlling covariates were retained at *P* = 0.1.

**Figure 1:**
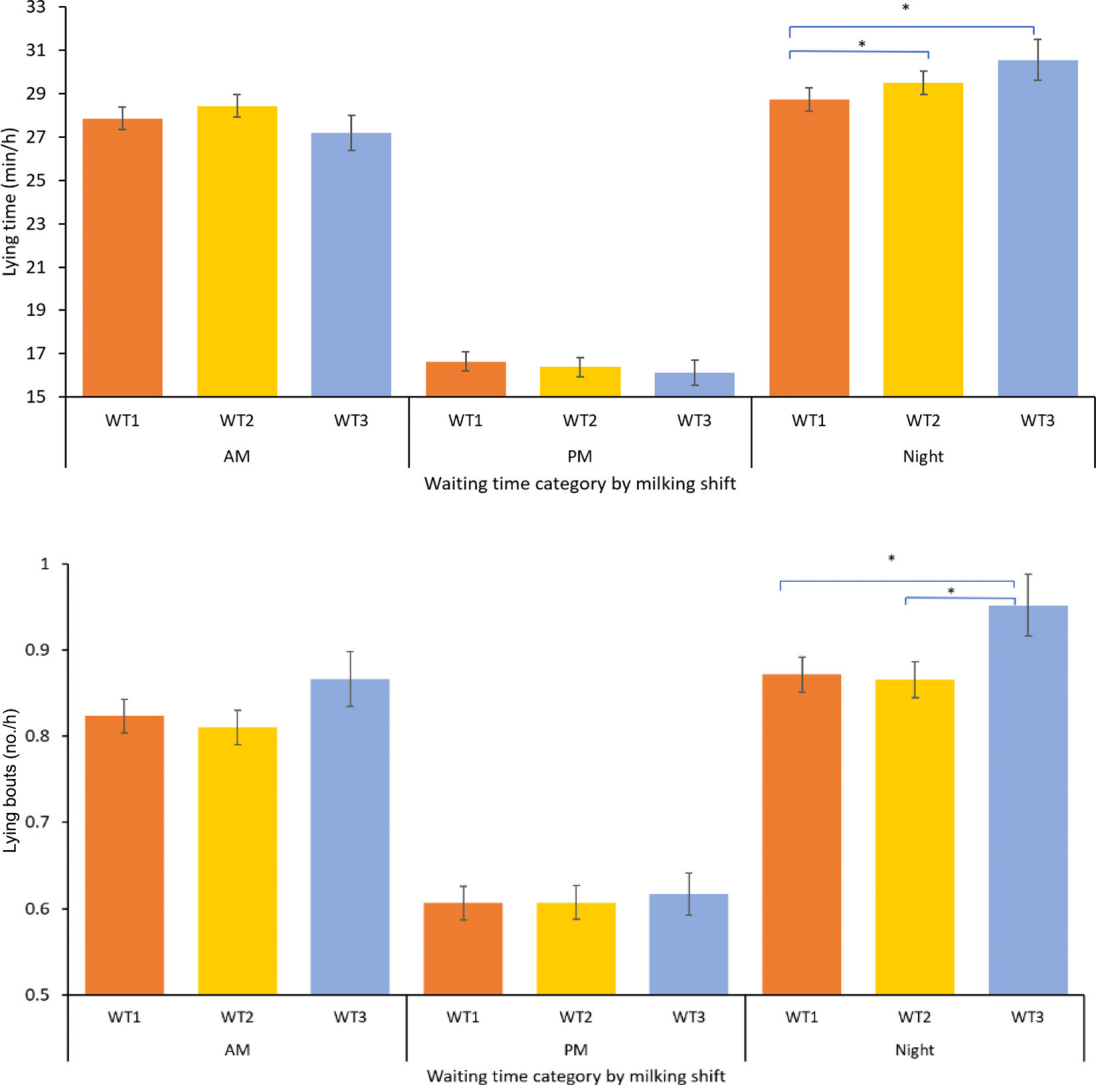
Least squares means (SEM bars) for lying time (top panel) and lying bouts (bottom panel) and number of steps (bottom panel) by premilking waiting time (WT) category during the 3 daily milkings: AM (0700 h), PM (1500 h), and night (2300 h). Asterisks indicate *P* < 0.05 for comparisons between WT categories.

**Figure 2:**
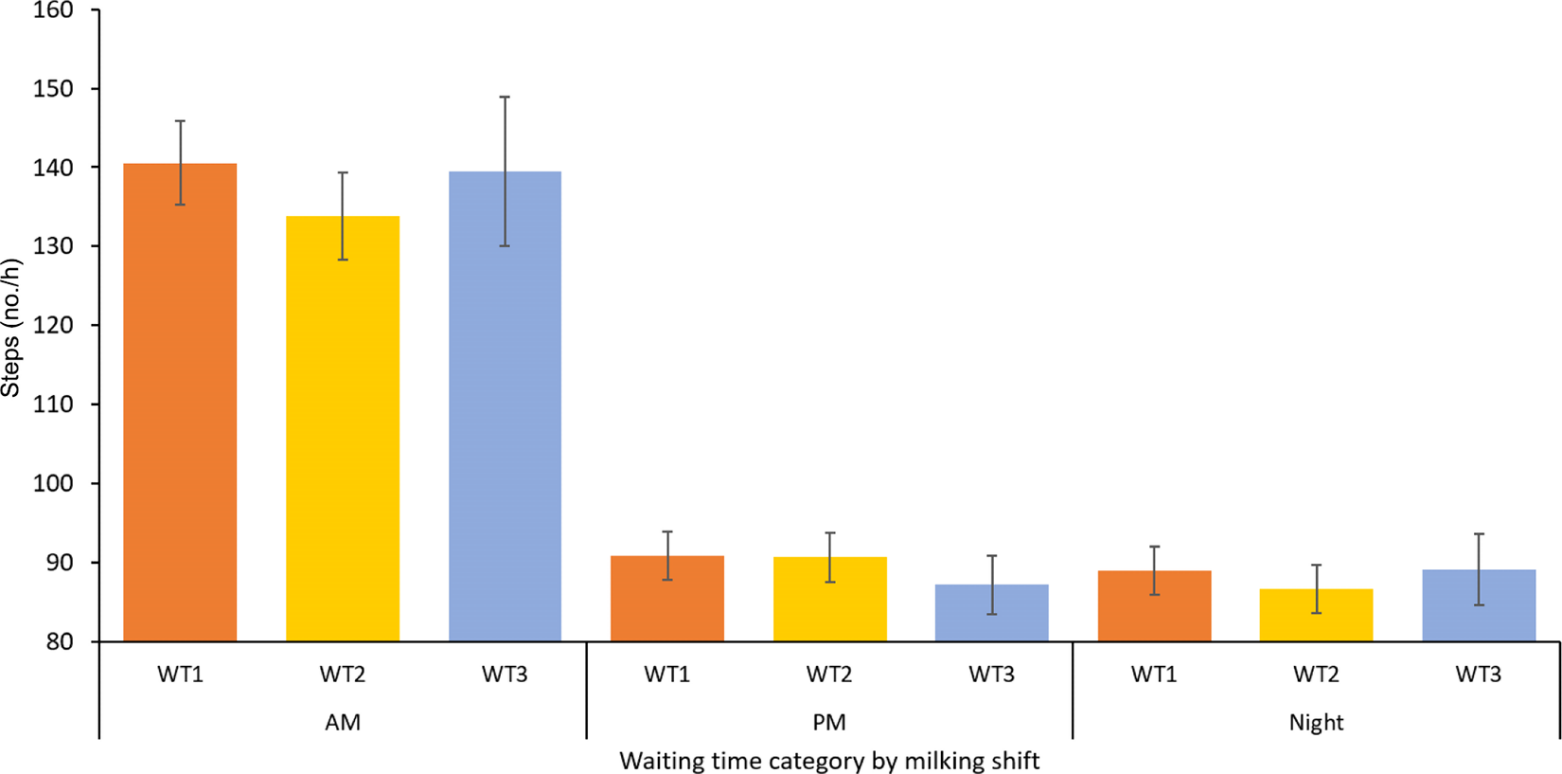
Least squares means (SEM bars) for number of steps by premilking waiting time (WT) category during the 3 daily milkings: AM (0700 h), PM (1500 h), and night (2300 h).

The average size of the milking group reported by the dairy farm was 335 cows. A total of 11,081 milking records were analyzed. The overall time (mean ± SD) between milkings during the observation period was 8.0 h ± 38 min, whereas the overall WT was 30.6 ± 23.4 min. Regarding lying and walking behaviors, overall LT, LB, and STP were 24.8 ± 10.9 min/h, 0.8 ± 0.39 bouts/h, and 93.4 ± 81.3 steps/h, respectively. Overall, 50.9% (n = 5,644), 43.4% (n = 4,811), and 5.7% (n = 626) of the WT were categorized as WT1, WT2, and WT3 during the monitoring period, respectively, and 62.0% of the milkings were classified as occurring at high THI (>40).

Table 1 summarizes the overall behavior budgets between 2 consecutive milkings for each study outcome stratified by level of the explanatory variables considered in the analysis. No significant effect for WT category on LT was established. Only cows in WT2 tended (*P* = 0.08) to exhibit greater LT compared with cows in WT1 (Table 1). On the other hand, differences were determined for LT behavior following different milking shifts, and low THI (≤40) was associated with reduced LT during the study period (Table 1). Neither parity nor health had a significant effect on LT. When LT was analyzed by milking shift, differences were only observed following the night shift, in which cows in WT1 had lower LT compared with cows in WT2 and WT3 (Figure 1).

**Table 1:** Effects of premilking waiting time (WT), milking shift, parity, and THI on lying and walking behavior budgets (LSM ± SE) after milking

Variable	Lying time (min/h)	*P*-value	Lying bouts (no./h)	*P*-value	Steps (no./h)	*P*-value
Waiting time (WT)						
WT1 (≤30 min)	24.4 ± 0.28	Referent	0.78 ± 0.01	Referent	106.4 ± 2.91	Referent
WT2 (30–60 min)	24.8 ± 0.29	0.08	0.76 ± 0.01	0.71	103.4 ± 3.02	0.39
WT3 (>60 min)	24.6 ± 0.45	0.85	0.80 ± 0.02	0.08	109.4 ± 4.80	0.76
Milking shift^1^						
AM	27.8 ± 0.53	Referent	0.83 ± 0.02	Referent	139.2 ± 5.60	Referent
PM	16.9 ± 0.44	<0.0001	0.62 ± 0.02	<0.001	90.6 ± 3.15	<0.0001
Night	29.5 ± 0.57	0.05	0.88 ± 0.02	0.03	89.3 ± 3.21	<0.0001
Parity						
Multiparous	24.7 ± 0.31	Referent	0.77 ± 0.01	Referent	92.4 ± 2.14	Referent
Primiparous	23.6 ± 0.89	0.25	0.88 ± 0.02	0.27	120.3 ± 5.13	<0.0001
THI category^2^						
High (>40)	26.2 ± 0.3	Referent	0.78 ± 0.01	Referent	112.2 ± 2.96	Referent
Low (≤40)	23.0 ± 0.32	<0.0001	0.76 ± 0.01	0.005	100.5 ± 3.00	<0.0001

1 Milking time: AM = 0700 h, PM = 1500 h, and night = 2300 h.

2 THI = temperature-humidity index.

Overall, WT category had no effect on LB, although a tendency for greater LB was determined for cows in WT3 (*P* = 0.08; Table 1). Milking shift had a significant effect on LB, as cows had the lowest and highest counts (number of bouts) after the PM and night milkings, respectively. Finally, cows exposed to high THI had greater LB than cows under low THI. When the association between WT and LB was analyzed by milking shift, cows in WT3 had greater LB counts than cows in WT1 and WT2 during the night shift (Figure 1).

Finally, WT was not associated with STP after milking. As shown in Table 1, milking shift was associated with STP, with the greatest STP occurring after the AM milking. The effect of parity category on STP was also significant and indicated that PP cows had greater numbers of steps than MP cows. Cows under high THI had greater numbers of steps than cows in cooler conditions.

The overall WT of the study cows was characterized for a large standard deviation and a coefficient of variation of 0.76, indicating that time in the holding area is highly variable among cows. A recent study by McVey et al. (2020) reported that cows were consistent in their milking order and, consequently, they would be consistent in their WT. In this study, we observed a large variability in WT, and it might be interesting to determine the level of consistency for individual cows over extended periods. To analyze this situation, entropy analyses have been suggested, which can confirm whether the observed hierarchies come from acquired behaviors or from randomness (McVey et al., 2020).

Appropriate daily routines and human–animal interactions, together with adequate stocking density, are crucial for successful dairy operations and can affect premilking waiting times (Hemsworth, 2003; Manriquez et al., 2018). In this study, stocking density was consistent as cows transitioned from the fresh pen (80%) to the subsequent groups (100%). However, our analysis did not consider the variation that multiple milker shifts could add to the variables in study.

In this study, cows showed specific lying and walking behaviors after each milking shift (Table 1), which could be associated with management tasks completed at different times of the day, different ambient conditions (such as THI), and the inherent daily cycle of cows (Kendall et al., 2008).

Lying budgets presented in this study are similar to those previously reported in the United States, where Holstein cows spent 9 to 10 h/d lying down (Ito et al., 2014).

Differences in LT among WT categories were only observed following the night shift (Figure 1). Although the magnitude of these differences was small, this finding suggests that an extended WT can influence subsequent behaviors, because cows waiting more than 30 min favored LT, which is considered a resting behavior (Dittrich et al., 2019). The reduced LT observed following the PM milking shift could be associated with the biological daily cycle in cows and with specific farm management tasks, such as feed delivery (Munksgaard et al., 2005). Additionally, the greater frequency of LB in cows subjected to longer WT in the night milking may reflect a higher level of cows' discomfort as they arrive to a pen crowded by cows, which may restrict opportunities for finding a resting stall. However, other complex factors, such as social hierarchy, are likely affecting the associations between WT and behavior and should be examined using more complex techniques in studies especially designed for that objective (McVey et al., 2020).

In contrast to other studies reporting lower LT in PP than in MP cows during transition (Kaufman et al., 2016; Silper et al., 2017; Succu et al., 2020), we did not observe an effect of parity on lying behavior. However, we determined that MP cows have a greater frequency of LB compared with PP, which is also in contradiction to other studies (Kaufman et al., 2016; Silper et al., 2017). Nevertheless, we observed a greater number of STP in PP, which concurs with previous reports establishing that PP cows are more active than older cows. However, our results were limited by the small number of PP cows in our study.

Ambient conditions play a significant role in the performance and behavior of lactating cows (Stone et al., 2017; Succu et al., 2020). We observed that THI modified the locomotion behavior of the study cows. Studies have shown that THI affects LT and the activity of dairy cows, and THI impacts MP and PP cows differently (Stone et al., 2017), likely because of the baseline difference in behavior budgets between growing and mature dairy cows.

In this study, we determined a small effect of WT on lying behaviors after the night milking. Variables such as parity, THI, and time of day affected cow behavior and should be considered when evaluating the impact of routine management tasks, such as milking. Although the associations identified in this research might be extrapolated to other dairies, our results are limited to the specific setting associated with organic milk production with its unique management requirements.
